# Identification of type of threading dislocation causing reverse leakage in GaN p–n junctions after continuous forward current stress

**DOI:** 10.1038/s41598-022-05416-3

**Published:** 2022-01-27

**Authors:** Tetsuo Narita, Masakazu Kanechika, Jun Kojima, Hiroki Watanabe, Takeshi Kondo, Tsutomu Uesugi, Satoshi Yamaguchi, Yasuji Kimoto, Kazuyoshi Tomita, Yoshitaka Nagasato, Satoshi Ikeda, Masayoshi Kosaki, Tohru Oka, Jun Suda

**Affiliations:** 1 Toyota Central R&D Labs., Inc., Nagakute, 480-1192 Japan; 2 Institute of Materials and Systems for Sustainability (IMaSS), Nagoya University, Nagoya, 464-8601 Japan; 3 MIRISE Technologies Corporation, Nisshin, 470-0111 Japan; 4 Toyoda Gosei Co., Ltd., Ama, Aichi 490-1207 Japan; 5 Department of Electronics, Graduate School of Engineering, Nagoya University, Nagoya, 464-8603 Japan

**Keywords:** Applied physics, Electronic devices, Electronic and spintronic devices

## Abstract

Power devices are operated under harsh conditions, such as high currents and voltages, and so degradation of these devices is an important issue. Our group previously found significant increases in reverse leakage current after applying continuous forward current stress to GaN p–n junctions. In the present study, we identified the type of threading dislocations that provide pathways for this reverse leakage current. GaN p–n diodes were grown by metalorganic vapor phase epitaxy on freestanding GaN(0001) substrates with threading dislocation densities of approximately 3 × 10^5^ cm^−2^. These diodes exhibited a breakdown voltage on the order of 200 V and avalanche capability. The leakage current in some diodes in response to a reverse bias was found to rapidly increase with continuous forward current injection, and leakage sites were identified by optical emission microscopy. Closed-core threading screw dislocations (TSDs) were found at five emission spots based on cross-sectional transmission electron microscopy analyses using two-beam diffraction conditions. The Burgers vectors of these dislocations were identified as [0001] using large-angle convergent-beam electron diffraction. Thus, TSDs for which ***b*** = 1***c*** are believed to provide current leakage paths in response to forward current stress.

## Introduction

Vertical power devices based on gallium nitride (GaN) have recently been developed^1–8^ as a means of minimizing power losses in electrical conversion systems requiring high current capabilities, such as automotive applications^9,10^. This technology is expected to make a significant contribution to future sustainable development initiatives. It is important for electrical systems, especially those used in automotive applications, to be reliable, and the reliability of vertical GaN p–n junctions subjected to high reverse biases, temperatures and current injections has been assessed. Because electric field crowding at device edges significantly lowers the breakdown voltage and can cause irreversible breakdown, various types of edge terminations have been developed and have demonstrated good avalanche capabilities^7,8,11–16^.

In power devices, the reverse leakage current below the breakdown voltage needs be controlled. The previous studies discussed the impacts of the threading dislocations as sources of reverse leakage current, focusing on the initial reverse bias characteristics^17–21^. Compared with the number of studies regarding the initial reliability of these devices, there have been few reports concerning hold tests under high reverse bias or high current stress^22–24^. Kizilyalli et al.^22^ subjected p–n diodes on freestanding GaN substrates having threading dislocation densities in the range of 10^4^ to 10^6^ cm^−2^ to high temperatures and reverse biases and found that the majority of the diodes exhibited a durability of over 1000 h. Our group also demonstrated that GaN p–n junctions with threading dislocation densities less than 10^4^ cm^−2^ were not degraded even under a high reverse bias corresponding to the avalanche condition at 175 °C over time spans of up to 1 h^24^. However, in contrast to this highly robust behavior, the reverse leakage current in some diodes rapidly increased after continuous forward current stress was applied^22–24^. This degradation mode could be a significant issue in metal–oxide–semiconductor field-effect transistors because, in such devices, a power circuit turns on the body p–n diode to allow a reflux current to flow during the switching operation. In a previous study, we found the degradation induced by a forward current occurred locally and might originate from threading dislocations^24^, although the specific defects in the current leakage path were not determined. Identifying these defects is very important for the further improvement of GaN bulk substrates because, while the elimination of all defects would be extremely difficult, the reduction of one type of defect may be feasible.

In the present work, we performed cross-sectional transmission electron microscopy (TEM) analyses of threading dislocations extracted from current leakage paths induced by forward current stress. We also identified the Burgers vector of the dislocations most frequently found at the leakage paths, using the large-angle convergent-beam electron diffraction (LACBED) method^25,26^.

## Methods

**Figure 1:**
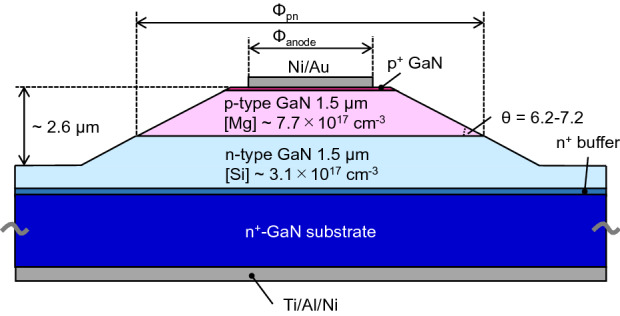
Diagram of a vertical p–n diode.

The layered structures of the experimental p–n junctions were grown using metalorganic vapor phase epitaxy in conjunction with commercially available freestanding GaN(0001) substrates prepared via hydride vapor phase epitaxy. The typical threading dislocation density in these specimens was 3 × 10^5^ cm^−2^ based on the etch pit density using the fused alkali. As shown in Fig. 1, the layered structure was composed of (from top to bottom) a 0.1 μm-thick p^+^-type GaN contact layer doped with magnesium (Mg) at a concentration of 5 × 10^19^ cm^−3^, a 1.5 μm-thick p-type GaN layer doped with Mg at a concentration of 7.7 × 10^17^ cm^−3^, a 1.5 μm-thick n-type GaN layer doped with silicon (Si) at a concentration of 3.1 × 10^17^ cm^−3^ and an n^+^-type GaN buffer layer with a Si concentration of 1.8 × 10^18^ cm^−3^. The surface electric field was reduced by forming a bevel mesa through the p-type layer at an angle of 6.2° to 7.2° by inductively coupled plasma reactive ion etching^12,27^. A nickel/gold (Ni/Au) metal stack was deposited as an anode electrode and was subsequently sintered at 500 °C under oxygen to form the Ohmic contact. Finally, a titanium/aluminum/Ni (Ti/Al/Ni) cathode electrode was formed on the reverse side of the substrate. In the present study, we focus on the characterization of three representative diodes with different diameters. Specifically, the anode and junction diameters (Φ_anode_ and Φ_pn_) were 60 and 184 μm for diode #1AX, 220 and 342 μm for #1CX, and 320 and 442 μm for #1DX.

The electrical properties of the specimens were sequentially characterized. Reverse bias sweeps up to the breakdown voltage were conducted at 25, 100 and 175 °C, followed by 1 h hold tests at 80% of the breakdown voltage at these respective temperatures. Hold tests performed for a duration of 1 h at a constant current of 1 mA, which corresponded to the avalanche condition, were subsequently carried out at the same temperatures. Following this, continuous forward current stress was applied by increasing the current density in a stepwise manner. The current density, *J*, during these stress trials was 50, 100, 200 and 500 A/cm^2^ (normalized by the anode area) and was each applied for 1 h. Those diodes that had exhibited increased reverse leakage current during the forward current tests were observed by emission microscopy (EMS) using an InGaAs detector sensitive over the wavelength range of 900 to 1600 nm. Defect-related carrier recombination sites associated with local leakage paths appeared as luminous points in the resulting EMS images^28^. It should be noted that these EMS observations were performed from the backside of the device after removing the cathode electrode by polishing. TEM specimens having ($1\overline{1}00$) cross-sections were fabricated near the luminous points and 0002 and $11\overline{2}0$ two-beam diffractions were assessed. The LACBED analyses were performed using a JEOL JEM-ARM300F at an acceleration voltage of 300 kV. This technique allowed the determination of the Burger vectors associated with dislocations based on analyzing the interactions between high-order Laue zone lines and a threading dislocation line^25,26^.

## Results and discussion

**Figure 2:**
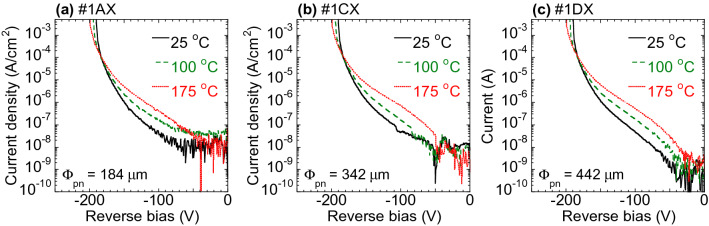
Reverse bias characteristics of the representative (**a**) #1AX, (**b**) #1CX and (**c**) #1DX diodes at 25, 100 and 175 °C. The current density values have been normalized by the area of the p–n junction, and the junction diameters, Φ_pn_, are provided at the base of each figure.

Figure 2 presents the current density–voltage (*J*–*V*) curves acquired in conjunction with reverse biases at 25, 100 and 175 °C for three representative diodes. Note that these *J* data have been normalized by the junction areas. The breakdown voltages at 25 °C were in the range of 191–192 V, and so were close to the ideal avalanche breakdown voltage of 174 V expected from the doping concentration in these devices^29,30^. The breakdown voltages were found to increase with increasing temperature, which is characteristic of avalanche breakdown^12,27^. All diodes exhibited repeatable breakdown characteristics, indicating good avalanche capabilities owing to the shallow beveled mesa termination^12,27^. The leakage currents and avalanche voltages for all diodes were stable throughout each 1 h hold test both at 80% of the breakdown voltage and under avalanche conditions at 25, 100 and 175 °C (see Supplementary Information). These results were in keeping with those of a previous study^24^ using p–n diodes fabricated on freestanding GaN substrates having threading dislocation densities less than 10^4^ cm^−2^. It is therefore evident that threading dislocations had a minimal effect on the robustness of such devices when subjected to reverse bias stress.

**Figure 3:**
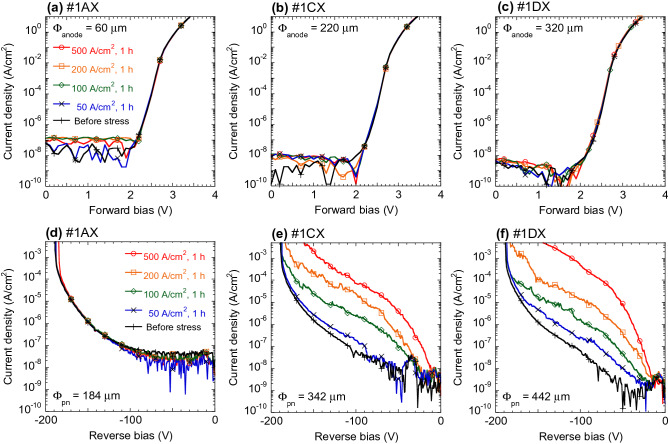
(**a**)–(**c**) Forward and (**d**)–(**f**) reverse current density–voltage curves acquired during continuous current injection tests. The injected current density values have been normalized by the anode areas in (**a**)–(**c**), and anode diameters, Φ_anode_, are provided at the top of each figure. The reverse leakage current density values in (**d**)–(**f**) have been normalized by the area of the p–n junction, and the junction diameters, Φ_pn_, are provided at the base of each figure.

**Figure 4:**
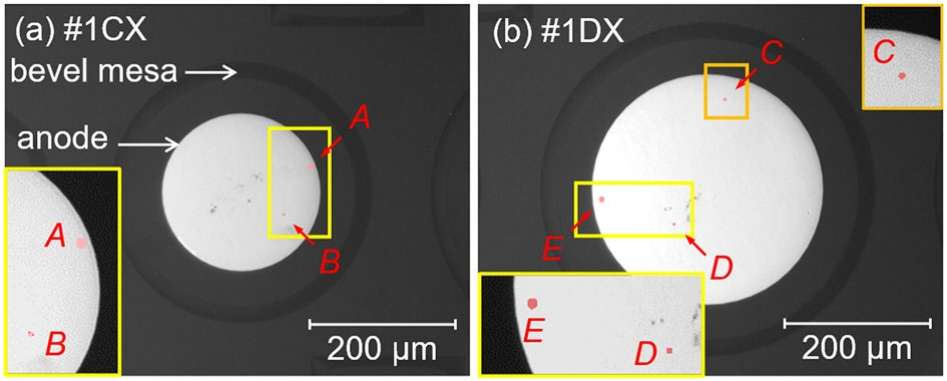
(**a**) (**b**) EMS images of diodes #1CX and #1DX. The inserted images are magnifications of areas near the respective luminous points. In the case of diode #1CX shown in (**a**), two luminous points, *A* and *B*, were observed in conjunction with a reverse leakage current of 500 nA at –120 V. For diode #1DX shown in (**b**), three luminous points, *C*, *D* and *E*, appeared at a reverse leakage current of 400 nA at –78 V.

Figure 3 shows the forward and reverse bias characteristics of the specimens during continuous forward current trials. Diode #1AX, which had the smallest anode diameter (Φ_anode_ = 60 μm), showed constant forward and reverse bias properties during the test period. We also confirmed that the other two diodes (with Φ_anode_ = 60 and 120 μm) were similarly stable (data not shown). In contrast, the leakage current levels observed in the case of diodes #1CX and #1DX (with respective Φ_anode_ values of 220 and 320 μm) rapidly increased with increasing *J* stress. Similar degradation was demonstrated by the other three diodes with Φ_anode_ values of more than 220 μm (data not shown). Figure 4a and b provide EMS images for diodes #1CX and #1DX acquired after continuous *J* stress tests at reverse biases of − 120 and − 78 V, respectively. Since the EMS observations were performed from the backside of each device, these images have been flipped horizontally so as to present the views from the anode side. Two luminous points, *A* and *B*, are apparent in the image of diode #1CX, while three EMS points, *C*, *D* and *E*, can be seen in that of diode #1DX. Based on the average threading dislocation density of 3 × 10^5^ cm^−2^, 110 and 240 threading dislocations would be expected to be present under the anode electrodes in samples #1CX and #1DX, whereas only two and three luminous points are apparent in the EMS images, respectively. In addition, the diodes with Φ_anode_ = 60 and 120 μm that showed no degradation would be expected to contain 8.5 and 34 threading dislocations under the anode electrodes, respectively. These results suggest that only a few threading dislocations provided reverse current leakage paths during the continuous *J* tests, while the reverse leakage current at the majority remained at the low level as before experiencing the forward current stress.

**Figure 5:**
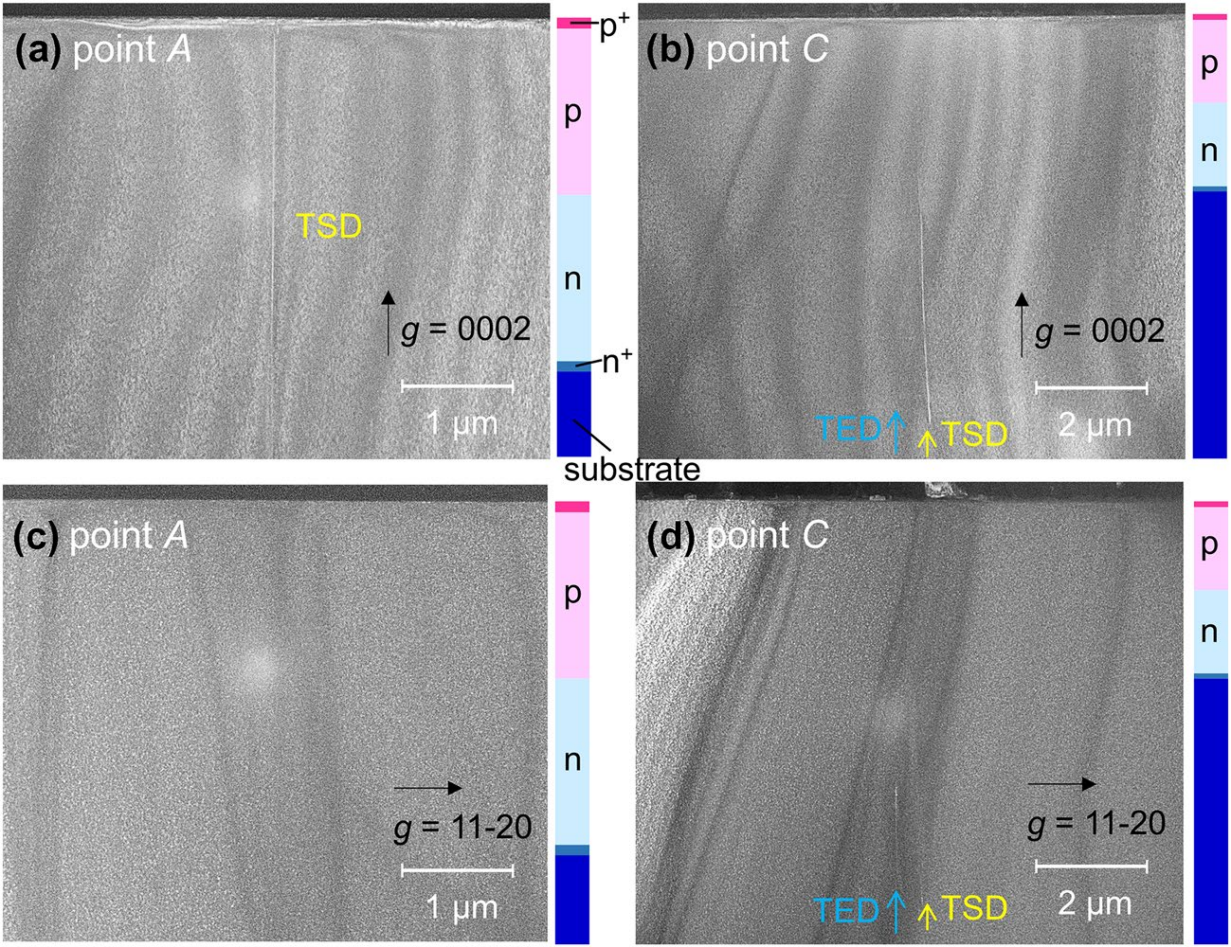
Dark-field TEM images of (**a**) (**c**) luminous point *A* in diode #1CX and (**b**) (**d**) luminous point C in diode #1DX. The diffraction conditions for (**a**) (**b**) and (**c**) (**d**) were ***g*** = 0002 and ***g*** = $11\overline{2}0$, respectively. The schematic on the right side of each figure indicates the layer structure.

Figure 5 shows dark-field cross-sectional TEM images acquired at luminous points *A* and *C*. The ***g*** = 0002 and ***g*** = $11\overline{2}0$ diffraction conditions allowed the visualization of threading dislocations having screw and edge components, respectively. In the case of luminous point *A*, one threading dislocation visible only for the 0002 diffraction condition was detected and was assigned to a closed-core threading screw dislocation (TSD). For luminous point *C*, one closed-core TSD along with a threading edge dislocation (TED) was observed. Both threading dislocations at point *C* passed through the p–n junction, although they disappeared near the junction as a consequence of the thinning process applied to the TEM specimen. A TSD passing through the junction was found at each of EMS points *B*, *D* and *E* in the degraded diodes #1CX and #1DX, as summarized in Table 1. We also performed a TEM analysis for the EMS point previously examined^24^ in degraded p-n diodes on an acidic ammonothermal-grown freestanding GaN substrate having a threading dislocation density of less than 10^4^ cm^−2^ and found a TSD (data not shown). As summarized in Table 1, we detected the common TSDs at eleven EMS points for p–n diodes on two different types of GaN substrates. These results suggest that closed-core TSDs provided leakage pathways after the application of forward current stress. In the p–n diodes of the present study, the portion of TSDs to the total threading dislocations is unknown because it is not easy to separate TSDs from the threading mixed dislocations (TMDs) even using the etch pit methods^31^. In general, the ratio of the number of TSDs to the total number of threading dislocations in freestanding GaN substrates will be relatively small^31–33^. This may explain the robust nature of some p-n diodes incorporating threading dislocations and the small number of luminous points compared with the total number of threading dislocations seen in the EMS images after continuous *J* stress.

**Table 1:** Summary of threading dislocations observed near each EMS point.

Type of substrate	Diode ID	Anode diameter	EMS point	Type of threading dislocation
HVPE threading dislocation density ~ 3 × 10^5^ cm^-2^	#1CX	220 μm	*A*	One TSD (***b*** = 1***c***)
			*B*	One TSD
	#1DX	320 μm	*C*	One TSD and one TED
			*D*	One TSD (***b*** = 1***c***)
			*E*	One TSD and two TEDs
	#2CX	220 μm	*I*	One TSD
			*J*	Not examined
Acidic ammonothermal^24^ threading dislocation density $<$ 10^4^ cm^-2^	#2DX	320 μm	*V*	One TSD
	#3FX	520 μm	*W*	One TSD
	#1FX	520 μm	*X*	One TSD
			*Y*	One TSD (***b*** = 1***c***)
			*Z*	One TSD (***b*** = 1***c***)

The p-n diodes on two different types of GaN substrates were examined. The HVPE substrate was used in the present study, while the acidic ammonothermal GaN substrate having a threading dislocation density less than 10^4^ cm^-2^ were examined in the previous study^24^. TEM analyses were employed eleven emission points for p-n diodes on the different two types of substrates, and detected at least one TSD at each EMS point. LACBED analyses were performed for four EMS points (*A*, *D*, *Y* and *Z*) and gave a common result of ***b*** = 1***c.***

**Figure 6:**
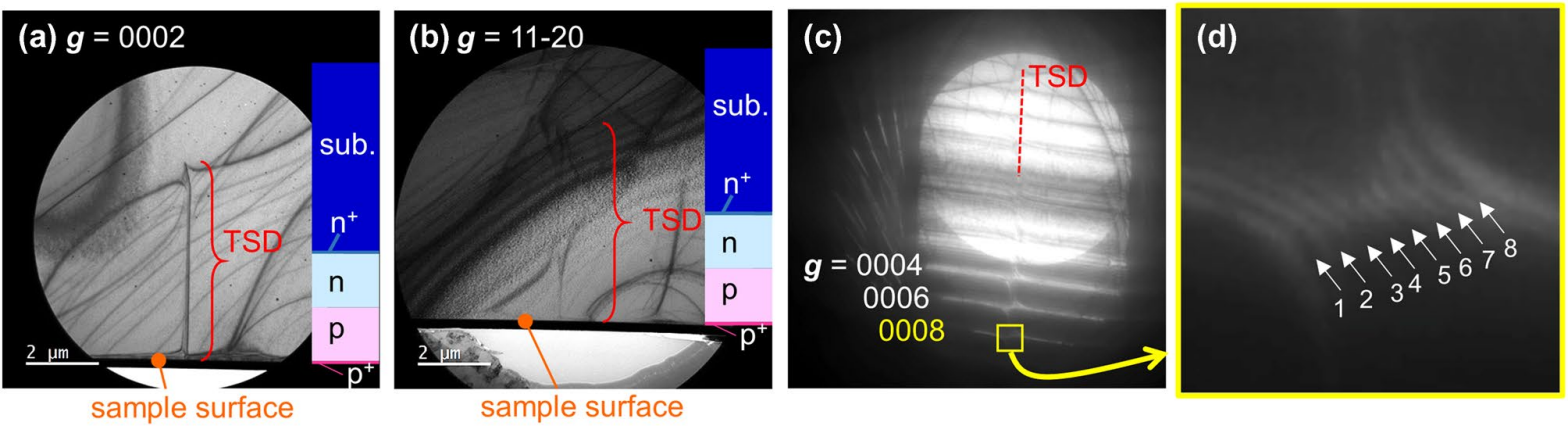
Bright-field TEM images of the TSD at EMS point *D* in diode #1DX with (**a**) ***g*** = 0002 and (**b**) ***g*** = $11\overline{2}0$. Note that the sample surface was directed downward. (**c**) Dark-field LACBED image of the TSD (denoted by the red dashed line). (**d**) Magnified LACBED pattern around ***g*** = 0008. The white arrows indicate eight split lines resulting from the interaction of the Laue zone line for ***g*** = 0008 and the TSD line.

LACBED analyses were performed to identify the Burgers vectors, and Fig. 6a and b show the two-beam bright-field TEM images obtained from luminous point *D*, which indicate the existence of one TSD. The Burgers vector for this TSD can be expressed as ***b*** = [000*x*], and Fig. 6c and d present LACBED images for this dislocation. It should be noted that for higher order diffractions ***g*** can interact with a threading dislocation line, to split into multiple lines whose number is $n = {\varvec{g}}\cdot {\varvec{b}}$ ^25,26^. Figure 6d shows the magnified LACBED pattern near ***g*** = 0008 and exhibits eight split lines (*n* = 8). We were therefore able to determine the Burgers vector for the TSD associated with point *D* as ***b*** = [0001]. The same analysis was performed for the TSD at emission point *A* and for the TSD at emission points *Y* and *Z* in the previously examined diode on the acidic ammonothermal-grown GaN substrate having the threading dislocation density less than 10^4^ cm^−2^, and the identical conclusions of ***b*** = [0001] were obtained. These results suggest that simple TSDs having ***b*** = 1***c*** provided leakage paths after the continuous *J* stress tests although we cannot say all TSDs with b = 1***c*** being the leakage pathways due to the small number of LACBED examinations at the present. In previous work using p–n diodes with much lower threading dislocation densities, there was good agreement between EMS points acting as leakage paths and the threading dislocations in the topographic images before the stress test^24^. This indicates TSDs were not mobile during the *J* stress test. Furthermore, other types of threading dislocations were not observed near the leakage spots in Ref.^24^. Therefore, we propose that interactions between threading dislocations involving motion during the *J* stress tests were unlikely. Nakano et al.^34^ theoretically suggested that complexes formed from Mg atoms and TSDs can act as current leakage paths in p-type GaN. In the case that current injection promotes Mg diffusion along TSDs, Nakano’s suggestion could explain the present degradation mode, although further work is required to identify the conduction mechanism at the TSDs.

## Conclusions

Threading dislocations thought to act as current leakage paths after continuous forward current tests in GaN p–n junctions were extracted and characterized. Trials with p–n diodes fabricated on freestanding GaN substrates with threading dislocation densities of approximately 3 × 10^5^ cm^−2^ showed that the leakage current was increased for devices with Φ_anode_ values of more than 220 μm during forward current injection, whereas diodes having a Φ_anode_ of 60 μm were generally stable. EMS observations from the backside of each device identified the current leakage paths and the same closed-core TSDs were identified near each luminous point in these images. The Burgers vector of the extracted TSDs was identified as ***b*** = [0001]. The results of this work suggest that simple TSDs at least with ***b*** = 1***c*** provided significant leakage pathways in response to excitation during the continuous forward current stress trials.

## Supplementary Information

Supplementary Information.

## Data Availability

The data that support the findings of this study are available from the corresponding author upon reasonable request.
