# Supplementary material for: Identification of type of threading dislocation causing reverse leakage in GaN p–n junctions after continuous forward current stress
Source: Sci Rep. 2022 Jan 27;12:1458. doi: 10.1038/s41598-022-05416-3 (PMC8795273; doi:10.1038/s41598-022-05416-3)
Supplement: Supplementary file 1 — Supplementary Information. [file 41598_2022_5416_MOESM1_ESM.pdf]

Identification of type of threading dislocation causing reverse leakage in GaN p-n junctions after continuous forward current stress

Tetsuo Narita[1*], Masakazu Kanechika[2], Jun Kojima[2], Hiroki Watanabe[3], Takeshi Kondo[2], Tsutomu Uesugi[2], Satoshi Yamaguchi[1], Yasuji Kimoto[1], Kazuyoshi Tomita[2], Yoshitaka Nagasato[3], Satoshi Ikeda[3], Masayoshi Kosaki[4], Tohru Oka[4], and Jun Suda[2,5]

[1] Toyota Central R&D Labs., Inc., Nagakute 480-1192, Japan

[2] Institute of Materials and Systems for Sustainability (IMaSS), Nagoya University, Nagoya 464-8601, Japan

[3] MIRISE Technologies Corporation, Toyota 470-0309, Japan

[4] Toyoda Gosei Co., Ltd., Ama, Aichi 490-1207, Japan

[5] Department of Electronics, Graduate School of Engineering, Nagoya University, Nagoya 464-8603, Japan

[*]corresponding author: tetsuo-narita@mosk.tytlabs.co.jp

Hold tests under reverse bias stress

Figures S1(a)-(c) present the data from hold tests at constant reverse biases equal to 80% of the breakdown voltages at 25, 100 and 175 °C. All three diodes with different junction diameters (Φ_{pn}) exhibited stable leakage current levels throughout each 1 h test period. These results were in good agreement with previous characterizations of p-n diodes fabricated on freestanding GaN substrates with much lower threading dislocation densities (less than 10^4 cm^{-2}).[19] Figures S1(d)-(f) show the results of reverse bias hold tests at a constant current of 1 mA and various temperatures that correspond to the avalanche conditions. The avalanche voltages were stable for all diodes within each test period of 1 h, in agreement with previous findings.[19] These data suggest that GaN p-n diodes are highly robust in response to reverse bias stress.

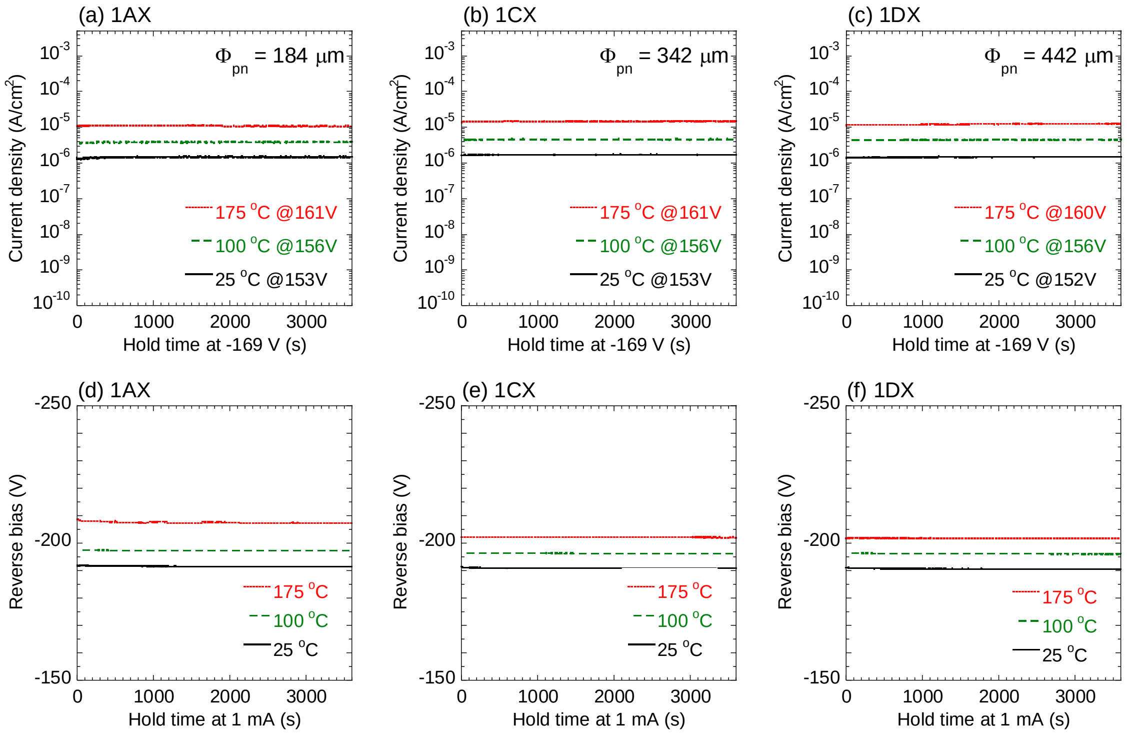

Figure S1. Current density as a function of time at a reverse bias of 80% of breakdown voltage at 25, 100 and 175 °C for diodes (a) #1AX, (b) #1CX, and (c) #1DX, with the junction diameter indicated at the top of each figure. Reverse bias as a function of stress time at a constant current of 1 mA and in the avalanche state for diodes (d) #1AX, (e) #1CX, and (f) #1DX.
